# Deaf children's non-verbal working memory is impacted by their language experience

**DOI:** 10.3389/fpsyg.2015.00527

**Published:** 2015-05-05

**Authors:** Chloë Marshall, Anna Jones, Tanya Denmark, Kathryn Mason, Joanna Atkinson, Nicola Botting, Gary Morgan

**Affiliations:** ^1^Department of Psychology and Human Development, UCL Institute of Education, University College LondonLondon, UK; ^2^Deafness, Cognition and Language Research Centre, University College LondonLondon, UK; ^3^Division of Language and Communication Sciences, City University LondonLondon, UK

**Keywords:** deafness, language, British Sign Language, working memory

## Abstract

Several recent studies have suggested that deaf children perform more poorly on working memory tasks compared to hearing children, but these studies have not been able to determine whether this poorer performance arises directly from deafness itself or from deaf children's reduced language exposure. The issue remains unresolved because findings come mostly from (1) tasks that are verbal as opposed to non-verbal, and (2) involve deaf children who use spoken communication and therefore may have experienced impoverished input and delayed language acquisition. This is in contrast to deaf children who have been exposed to a sign language since birth from Deaf parents (and who therefore have native language-learning opportunities within a normal developmental timeframe for language acquisition). A more direct, and therefore stronger, test of the hypothesis that the type and quality of language exposure impact working memory is to use measures of non-verbal working memory (NVWM) and to compare hearing children with two groups of deaf signing children: those who have had native exposure to a sign language, and those who have experienced delayed acquisition and reduced quality of language input compared to their native-signing peers. In this study we investigated the relationship between NVWM and language in three groups aged 6–11 years: hearing children (*n* = 28), deaf children who were native users of British Sign Language (BSL; *n* = 8), and deaf children who used BSL but who were not native signers (*n* = 19). We administered a battery of non-verbal reasoning, NVWM, and language tasks. We examined whether the groups differed on NVWM scores, and whether scores on language tasks predicted scores on NVWM tasks. For the two executive-loaded NVWM tasks included in our battery, the non-native signers performed less accurately than the native signer and hearing groups (who did not differ from one another). Multiple regression analysis revealed that scores on the vocabulary measure predicted scores on those two executive-loaded NVWM tasks (with age and non-verbal reasoning partialled out). Our results suggest that whatever the language modality—spoken or signed—rich language experience from birth, and the good language skills that result from this early age of acquisition, play a critical role in the development of NVWM and in performance on NVWM tasks.

## Introduction

Working memory is the capacity to encode, store, manipulate and recall information, and is essential for cognition (Baddeley and Hitch, 1974). As Hirshorn et al. (2012 p. 85) write, “One would be hard pressed to name any higher level cognitive ability that does not foundationally depend on holding information in memory and being able to manipulate and integrate it with knowledge from long-term memory.” Not surprisingly, therefore, individual differences in working memory are associated with variation in such diverse activities as reasoning ability (Kyllonen and Christal, 1990), the acquisition of computer programming skills (Shute, 1991), and a whole set of activities that require language, such as reading comprehension (Daneman and Carpenter, 1980), novel word learning (Kwok and Ellis, 2014), syntactic processing (King and Just, 1991), second language learning (Kormos and Sáfár, 2008), acquiring an artificial language (Kapa and Colombo, 2014), and even adjusting to non-native speakers' lexical reference (Lev-Ari, 2015). Furthermore, individual differences in children's working memory are closely linked to their academic achievement (Alloway et al., 2005; Engel de Abreu et al., 2014). In the recent literature, the term working memory has been used to describe only the complex and executive-loaded elements of memory, i.e., where concurrent maintenance and processing of information are required for task completion. The focus of the study reported in the current paper is the nature of the association between language and working memory in the wider sense, although we were particularly interested in the complex and executive-loaded tasks.

As is often the case when trying to understand the nature of associative relationships between cognitive variables, it is far from straightforward to establish causal direction, i.e., whether differences in working memory drive individual differences in language during development, or vice versa. Longitudinal studies of children's vocabulary size have suggested that working memory ability does indeed drive vocabulary development rather than the other way round (Avons et al., 1998). Mechanistically, the claim is that the phonological loop (a component of phonological working memory; Baddeley and Hitch, 1974) provides a temporary means of storing new words, before they are consolidated in phonological long term memory (Baddeley et al., 1998). However, the strength of working memory as a predictor of vocabulary size declines with age (Gathercole et al., 1992) and is not found in all studies (Melby-Lervag et al., 2012).

A window onto the question of whether the causal influence might also operate in the opposite direction, i.e., whether individual differences in language can drive differences in working memory, comes from deaf children whose language learning experience is very different from that of the vast majority of children. The incidence of significant congenital deafness is about 1 in 1000 live births in most developed countries, including the UK, although it may be 3–4 times higher in certain communities or parts of the UK (Davis et al., 1997). Even mild deafness (defined as a hearing loss of 21–40 decibels) can cause difficulties accessing spoken language and have a detrimental effect on linguistic development. Hearing aids and cochlear implant technology, while improving rapidly, do not offer access to the same quality of speech that hearing children obtain naturally (Faulkner and Pisoni, 2013).

Sign languages such as British Sign Language (BSL) *do* offer a fully accessible language form to deaf children who do not have co-occurring visual impairment, but the vast majority of deaf children (over 90%; Lederberg and Mobley, 1990) are born to hearing non-signing parents. This means that even in cases where hearing parents learn BSL and sign with their children from an early age, the quality and quantity of language input and interaction that they are able to provide is likely to be impoverished compared to that provided by deaf signing parents. Nevertheless, for deaf children born to deaf signing parents, who receive sign language input from birth, language acquisition can show remarkable parallels in onset, rate and patterns of development compared to hearing children who are learning spoken languages (see Chamberlain et al., 2000; Morgan and Woll, 2002; Schick et al., 2005 for reviews). Deaf children of deaf parents (i.e., native signers) are therefore a very interesting population theoretically, but they are also very difficult to recruit to research studies. Not only are there a very small number of children in this group, but measuring their skills requires carefully-designed tasks and a researcher fluent in the particular sign language under consideration (Lieberman and Mayberry, 2015).

The diversity of language input in the deaf population, both with respect to age of access to language (from birth, later in infancy/childhood) and language form (signed or spoken), allows researchers to investigate how individual differences in linguistic input can impact on working memory development. In the remainder of this introduction, we review studies that have investigated working memory in deaf adults and children, identify the gaps in that literature, and motivate our own study.

A theme in the research literature on deaf people's working memory to date is a division between two types of studies: those that have investigated memory for spoken material, and those that have studied memory for signed and/or non-linguistic visuo-spatial material. Measurement of working memory across modalities requires serious consideration. It cannot be assumed that performance on a task presented in two different modalities is directly comparable. Likewise it cannot be assumed that two tasks presented in the same modality are directly comparable. As we discuss below, both modality and the nature of the material affect recall in working memory tasks.

It is perhaps not surprising that studies where material is presented auditorally find poorer recall by deaf participants in comparison to hearing participants. For example, Fagan et al. (2007) studied deaf children aged 6–14 years who received a cochlear implant between the ages of 1 and 6 years. Group means on spoken forward and backward digit span tasks were significantly lower than the standardized mean, with half the sample scoring below 1 SD from the mean on the forward task and the majority scoring below 1 SD from the mean on the backward task. Furthermore, scores on both span tasks were moderately correlated with vocabulary comprehension and non-word/rare-word reading scores. In another study, Burkholder and Pisoni (2003) divided deaf cochlear-implant users (aged 8–9 years) into two groups, according to whether they used just oral language or whether they used total communication (i.e., using manual sign and lip reading strategies, in addition to speech), and compared them to a group of hearing children on spoken digit span tasks. Both deaf groups performed significantly more poorly than the hearing group. The digit span disadvantage for deaf participants has been found even when the task bypasses listening/speaking by being presented in written form (Parasnis et al., 1996), and when letters are used instead of digits (Wallace and Corballis, 1973).

A disadvantage for serially-presented linguistic material is also found when deaf participants undertake the digit span or letter span task in a sign language. Deaf native American Sign Language (ASL) signers recall on average only 5 ± 1 digits in forward tasks, compared to hearers who recall an average of 7 ± 2 digits (Boutla et al., 2004; Bavelier et al., 2006). Hall and Bavelier (2010, p. 54) have concluded that “speech-based representations are better suited for the specific task of perception and memory encoding of a series of unrelated verbal items in serial order through the phonological loop.” Conway et al. (2009) go further and propose the “auditory scaffolding hypothesis,” whereby one's experience with sound helps provide a scaffold for the development of those general cognitive abilities that are required for the representation of temporal or sequential patterns. However, Bavelier and colleagues' work shows that *hearing* English-ASL bilingual adults also show the same disadvantage for sign span compared to spoken span (Bavelier et al., 2008), which challenges the auditory scaffolding hypothesis because these individuals have had rich auditory input since birth. In any case, it is clear that performance on spoken serial recall tasks may not be directly comparable to performance on signed serial recall tasks.

For non-linguistic material that is *not* processed using the phonological loop, but which, like linguistic material, is serial in nature, deaf signers have been shown to have an advantage compared to other groups. Deaf adult signers have longer forward spans than hearing non-signers on the visuo-spatial Corsi Block Test (Geraci et al., 2008). Wilson et al. (1997) showed that the advantage for deaf signers over hearing non-signers in the Corsi Block Test was also evident in 8–10 year-old children. Evidence that the working memory advantage might arise from using sign language, rather than from being deaf, comes from studies by Capirci et al. (1998) and Parasnis et al. (1996). The former study demonstrated that hearing children who were taught sign language at school performed on non-verbal working memory tasks better after 1 year than hearing children who were taught a spoken language (Capirci et al., 1998), while the latter study found that deaf orally-educated children did not have an advantage over hearing children (Parasnis et al., 1996).

When serial recall of material is not the only requirement of the working memory task, or indeed is not required at all, then the pattern of results looks different again. Differences have *not* been found between deaf signers and hearing non-signers on complex span tasks, which rely on some sort of processing of material in addition to serial maintenance. However, the difficulty of complex span tasks means that to date in the deafness and sign language literature they appear to have only been carried out with adults (e.g., Boutla et al., 2004; Andin et al., 2013).

In summary, several recent studies have suggested that deaf children perform more poorly on working memory tasks compared to hearing children, but they have not been able to determine whether this poorer performance arises directly from deafness itself or from deaf children's reduced language exposure. The underlying cause of deaf children's poor task performance remains unresolved because findings come mostly from (1) tasks that are verbal as opposed to non-verbal (e.g., Burkholder and Pisoni, 2003; Fagan et al., 2007) and (2) deaf children who use spoken communication and who may therefore have experienced impoverished language input or have language development delay (e.g., Burkholder and Pisoni, 2003; Fagan et al., 2007; Figueras et al., 2008; Beer et al., 2011; Hintermair, 2013). Such a group may potentially perform differently on working memory tasks compared to deaf children who have been exposed to a sign language since birth from Deaf parents (and who therefore have native language-learning opportunities within a normal developmental timeframe for language acquisition). The role of age of language exposure in the wider neuro-cognitive abilities of deaf individuals has also been highlighted (Campbell et al., 2014). Moreover, studies using complex span tasks have not been reported, to the best of our knowledge, with deaf children. As mentioned earlier, recruiting and testing deaf children with a range of language experiences, and particularly those who are native signers, is a challenging task. However, doing so provides important contrasts which enable us to start unpacking the influences of auditory experience and language background.

A more direct, and consequently stronger, test of the relationship between type and quality of language exposure and working memory is therefore to use measures of non-verbal working memory and to compare hearing children with two groups of deaf signing children: those who have had native exposure to a sign language, and those who have experienced delayed acquisition and reduced quality of sign language input compared to their native-signing peers. This is exactly what we set out to do in the present study. If it is language experience rather than deafness that impacts on working memory, then native deaf signers should pattern like hearing children and both groups should perform better than non-native signers. Furthermore, scores on language tasks should correlate with working memory scores. If, however, it is lack of auditory experience that causes poor working memory, or if it is the case that comorbid memory difficulties occur with deafness, then both deaf groups should perform worse than the hearing group. If neither language experience nor deafness has an impact on working memory, then the three groups would not be expected to differ from one another, and no relationship should be found between language and working memory scores.

## Methods

### Participants

Twenty seven deaf children aged 6–11 years old (16 boys) were recruited. All had profound and/or severe hearing loss in both ears, with the majority (*n* = 24) being profoundly deaf in both ears. All had been born deaf (i.e., none had been deafened in early childhood by meningitis, for example, and therefore none had early access to auditory input). However, none had additional learning difficulties, according to teacher and/or parental report.

All 27 deaf participants used BSL regularly, but had different levels of exposure to BSL and different degrees of BSL use. Based on their exposure to, and use of, BSL, they were divided into two groups: native signers (*n* = 8) and non-native signers (*n* = 19). To be included in the native signer group, participants had to have at least one deaf parent (some also had one or more deaf siblings, but this was not a requirement for inclusion) and to have been exposed to BSL from their parent(s) since birth. In addition, the parents of these children had to report that BSL was the language in which their child preferred to communicate and was the language in which the child communicated with his/her deaf parent(s). Although not part of the selection criteria, the eight children in this group (5 boys) were all reported to mix regularly with deaf adults and either half or the majority of their friends were reported to be deaf. Please see Table 1 for further details.

**Table 1:** **Language background of deaf native signers**.

Code^*^	**Age**	**Deaf family members**	**Parent-child language preference**	**Child's language preference**	**Age of acquisition of BSL**	**Who did child learn BSL from?**	**Are child's friends deaf?**	**Does child mix with deaf adults?**	**Level of deafness in left ear**	**Level of deafness in right ear**	**Does child wear hearing aids?**	**Does child have a cochlear implant?**
B01	7;1	Parent(s)	BSL	BSL	From birth	Deaf parent(s)	Most deaf	Yes	Profound	Profound	Sometimes	No
G02	8;0	Parent(s)	BSL	BSL	From birth	Deaf parent(s)	Most deaf	Yes	Profound	Profound	Sometimes	No
G03	7;1	Parent(s) + sibling(s)	BSL	BSL	From birth	Deaf parent(s)	Equal deaf/hearing	Yes	Profound	Profound	Sometimes	No
B04	7;6	Parent(s) + sibling(s)	BSL	BSL	From birth	Deaf parent(s)	Most deaf	Yes	Profound	Profound	Sometimes	No
B05	8;1	Parent(s) + sibling(s)	BSL	BSL	From birth	Deaf parent(s)	Equal deaf/hearing	Yes	Profound	Profound	No	No
G06	8;11	Parent(s) + sibling(s)	BSL	BSL	From birth	Deaf parent(s)	Most deaf	Yes	Profound	Profound	Yes	No
B07	7;10	Parent(s) + sibling(s)	BSL	BSL	From birth	Deaf parent(s)	Equal deaf/hearing	Yes	Severe	Severe	Yes	No
B08	9;9	Parent(s) + sibling(s)	BSL	BSL	From birth	Deaf parent(s)	Most deaf	Yes	Profound	Profound	Sometimes	No

* *B, Boy and G, Girl*.

The remaining 19 deaf participants (11 boys) were considered to be non-native signers. This group was characterized by a later age of acquisition of BSL than the native-signer group (*M* = 2;11 years, *SD* = 2;2 years, range = 0;7–9;0 years), and the majority (*n* = 13) were reported to use sign-supported English (SSE) or spoken English alongside BSL as their preferred language and with their hearing parents. As Table 2 shows, this was a more heterogeneous group with respect to language background and current language use than the native-signer group, as is to be expected.

**Table 2:** **Language background of deaf non-native signers**.

Code^*^	**Age**	**Deaf family members**	**Parent-child language preference**	**Child's language preference**	**Age of acquisition of BSL (years)**	**Who did child learn BSL from?**	**Are child's friends deaf?**	**Does child mix with deaf adults?**	**Level of deafness in left ear**	**Level of deafness in right ear**	**Does child wear hearing aids?**	**Does child have a cochlear implant?**
G09	11;9	Older sibling	BSL	BSL	5	Deaf older sibling and school	Most deaf	Yes	Profound	Profound	Sometimes	No
G10	11;9	Twin sibling	BSL + spoken English	BSL + spoken English	4	School and speech therapist	Most deaf	Yes	Profound	Profound	No	Yes
G11	7;3	Other(s)	BSL	BSL + spoken English	0.8	Mother	Equal deaf/hearing	Yes	Profound	Profound	No	Yes
B12	10;4	None	BSL	BSL	0.6	Unknown	Equal deaf/hearing	Yes	Profound	Profound	Sometimes	Yes
B13	9;10	None	BSL	BSL	4	School	Equal deaf/hearing	No	Profound	Profound	No	No
B14	7;1	None	BSL	BSL	1	Parents and language aide	Most hearing	Yes	Profound	Profound	Not any more	No
B15	6;8	None	BSL	BSL	3	Mother	Equal deaf/hearing	Yes	Profound	Profound	No	Yes
B16	9;7	None	BSL	BSL	1	Parents; deaf and hearing adults	Most deaf	Yes	Profound	Profound	No	No
G17	9;4	None	SSE	SSE + BSL	2	School	Most deaf	No	Profound	Profound	Yes	No
G18	8;3	None	SSE	SSE + BSL	3	School	Most deaf	Yes	Severe	Severe	Yes	No
B19	8;6	None	SSE	SSE + BSL	6	School	Equal deaf/hearing	Yes	Profound	Profound	Yes	No
G20	10;7	None	BSL + spoken English	BSL	1.6	Mother and school	Equal deaf/hearing	Yes	Profound	Profound	No	Yes
B21	10;2	None	BSL + spoken English	BSL	2	Mother	Most deaf	Yes	Profound	Profound	Sometimes	No
B22	11;0	None	BSL + spoken English	SSE	3	Peripatetic teacher of the deaf	Most hearing	Yes	Profound	Profound	Yes	No
G23	9;5	None	BSL + spoken English	SSE + BSL	1	Parents and nursery	Most hearing	Yes	Profound	Profound	No	Yes
B24	11;10	None	BSL + spoken English	BSL + spoken English	4	School	Most deaf	No	Profound	Profound	Not any more	Yes
B25	11;8	None	spoken English	BSL	9	School	Most deaf	No	profound	profound	yes	yes
B26	6;9	None	spoken English	BSL + spoken English	3	School	Most deaf	Yes	severe	severe	yes	no
G27	10;11	None	spoken English	BSL + spoken English	1	Parents and school	Most deaf	Yes	profound	profound	no	yes

* *B, Boy; G, Girl*.

Twenty eight hearing participants of the same age—6 to 11 years (16 boys)—were also recruited. All were reported by parents/and or teachers to have no hearing difficulties or learning difficulties of any kind, and all had English as their first language.

The mean age of the deaf participants was 9;2 years (*SD* = 1;8), and of the hearing participants was 9;0 (*SD* = 1;5). There was no significant age difference between the deaf and hearing groups, *t*_(53)_ = 0.320, *p* = 0.751. However, the native signers (*M* = 8;0, *SD* = 0;11) were significantly younger than the non-native signers (*M* = 9;7, *SD* = 1;9), *t*_(25)_ = 2.391, *p* = 0.025, and marginally younger than the hearing participants, *t*_(34)_ = 1.802, *p* = 0.080. The non-native signer and hearing groups did not differ on age, *t*_(45)_ = 1.289, *p* = 0.204.

With respect to non-verbal reasoning, as measured by the matrix reasoning subset of the Wechsler Abbreviated Scale of Intelligence (WASI; Wechsler, 1999), both groups had mean T-scores in the normal range (mean = 50, *SD* = 10), *M*_deaf_ = 52.33 (*SD*_deaf_ = 10.57) and *M*_hearing_ = 55.79 (*SD*_hearing_ = 8.48), and the scores did not differ significantly from one another, *t*_(53)_ = 1.338, *p* = 0.186. Within the deaf group, the native signer subgroup (*M* = 62.25, *SD* = 7.01) had significantly higher T-scores than the non-native signers (*M* = 48.16, *SD* = 8.96), *t*_(25)_ = 3.954, *p* = 0.001, and marginally higher T-scores than the hearing group, *t*_(34)_ = 1.967, *p* = 0.057. The hearing group had higher T-scores than the non-native signers, *t*_(45)_ = 2.959, *p* = 0.005.

### Materials

#### Working memory tasks

Two working memory tasks, namely the Spatial Span Task (Wechsler and Naglieri, 2006) and the Odd One Out Span Task (Henry, 2001), were selected after piloting as they require a minimal amount of verbal instruction and only non-verbal responses (i.e., pointing).

The *Spatial Span Task* (from the Wechsler Nonverbal Scale of Ability, Wechsler and Naglieri, 2006) is a measure of visuo-spatial short-term working memory similar to the Corsi Block Test. A set of nine identical blue blocks is affixed to a white board in an unstructured array. The examiner can view a number on each of the blocks and is seated directly opposite to the child being tested. Children are instructed to tap a sequence of blocks in the same order as the examiner in the “forward” test, and in the reverse order in the “backward” test. Children are administered two trials for each sequence length, beginning with two blocks, ranging up to a span of nine. Two trials of each sequence length are administered, and the test is terminated once both trials of the same sequence length are failed. The task begins with two practice trials in both the spatial span forward and backward conditions to ensure that the child understands the task. One point is awarded for each sequence accurately repeated.

The *Odd One Out Span Task* (Henry, 2001) is a measure of executive-loaded visuo-spatial working memory. It is presented in PowerPoint and comprises 63 slides, each displaying a set of three shapes. On each of the slides, two of the shapes are identical, and one is slightly different: the “odd one out.” The examiner shows the child a slide and asks them to identify which shape is the odd one out. The child is instructed to try to remember the location of this shape. The following slide contains an empty grid with three boxes, and the child is asked to point to the empty box in the same location as the shape that they have just seen. After four single-item trials have been displayed, the child is shown two sets of shapes in a row. There then follows a slide with two empty grids, one on top of the other. The child is instructed to point to the empty boxes in the same location as the two “odd” shapes they have previously seen, in the same order that they were presented. If the child initially verbalizes or signs their answer (e.g., *left*, *middle*, etc.), they are reminded that they need to point to the location of the shape. Trial length increases sequentially in blocks of four with a maximum of six sets of shapes. Once the child makes two errors within a block, the test is terminated. The total number of trials correctly recalled is then calculated. Before the test begins, two practice trials are administered to illustrate the task procedure: a single-item and a two-item trial. Correct responses to the practice items are indicated to the child if they do not initially answer correctly.

#### Language tasks

We used three tests of language, of which two were new adaptations of existing measures. An adapted version of the *Expressive One Word Picture Vocabulary Test* (EOWPVT; Brownell, 2000) was used to test single word vocabulary production. The full test was initially administered as per the instruction manual. The children are presented with single pictures that test knowledge of primarily simple nouns (e.g., *train*, *pineapple*, *kayak*), but also some verbs (e.g., *eating*, *hurdling*), and category labels (e.g., *fruit*, *food*). After four practice items, the test begins at various starting points depending on the child's age. Eight items must be labeled correctly in succession, and the experimenter works backwards if necessary until the basal is achieved. The test finishes when the child gets six successive incorrect answers. The EWOPVT was developed in the USA and so a few pictures (*n* = 3) were substituted with alternative pictures to make the test more culturally relevant for children in the UK (e.g., *raccoon* → *badger*). In order that the EOWPVT could be used to assess the vocabulary of both hearing and deaf children who communicate in BSL, it was necessary to exclude a number of test items that do not exist in BSL (e.g., *cactus*, *banjo*, “*musical instruments”* as a collective term). This list of 15 excluded items was established by administering the test to three native signing Deaf adults who primarily communicated in BSL. These items were then deducted from the children's total raw scores.

The *BSL Narrative Production Test* (Herman et al., 2004) was designed to assess deaf children's (age 4–11 years) expressive language by eliciting a narrative in BSL. The child first watches a short, silent video (on a DVD) acted out by two deaf children. Participants are instructed to watch it carefully as they are going to be asked to tell the story once the video has finished. The experimenter leaves the room while the child watches the video and returns once it has finished. The experimenter asks the child to tell the story. The aim is to elicit a spontaneous story, so no further prompting is given other than asking, “is there anything else?” to check that the child has finished. The child's narrative is videotaped for subsequent scoring. The test is scored based upon three components: (1) the content of the story (i.e., the level of detailed information included in their narrative); (2) story structure (i.e., introducing the participants and setting the scene, reporting the key events leading to the climax of the story, and detailing the resolution of the story at the end); (3) aspects of BSL grammar (including use of spatial location, person and object classifiers and role shift). The narratives were scored by an experimenter who was fluent in BSL and had completed the training course required for administrators/coders of the test.

Hearing control group children were also tested on their narrative skills using the same video to elicit a spontaneous story in spoken English. As the original story is told only through gesture and action, this prompted the hearing children to use some gesture in their story retellings e.g., when describing the boy demanding food from the girl, a child may say: “Then he went like that [gestures putting out hand].” These gestures were included in the scoring of the story content. Because English and BSL grammar systems are very different, only narrative content and structure were scored for the purpose of this study. The reliability of the use of the test in spoken English was investigated with composite scores of structure and content. Twenty-four of the narratives were scored by two trained testers, showing good inter-rater reliability (*r* = 0.97, *p* < 0.001). Ten of the narratives were scored a second time by the same scorer, showing high intra-rater reliability (*r* = 0.98, *p* < 0.001). The internal consistency between the content and structure items of the measure was also high (*r* = 0.90, *p* < 0.001).

The *Language Proficiency Profile-2* (LPP-2; Bebko and McKinnon, 1993) is a questionnaire completed by a person who is familiar with the child's language skills. The aim is to provide an overall evaluation of linguistic and communicative skills of deaf children, regardless of the specific language or modality in which they communicate (i.e., BSL, signed supported English, spoken English, etc.). Most usually the parents, but occasionally the teacher (*n* = 3, all in the deaf group), of the children participating in this study completed this questionnaire. The LPP-2 comprises five categories: (1) Form: structure of the language e.g., single words/signs in the early stages, later developing into the ability to produce short narratives; (2) Use: functions of language i.e., to interact or gain the attention of others etc.; (3) Content: the type of objects, actions and relationships that exist in the child's communication e.g., referring to the existence/disappearance of objects, information about denial or causality etc.; (4) Reference: the ability of the child to describe events beyond the present context; and (5) Cohesion: how effectively the child adapts their communication to the listener e.g., modification of syntax to account for the perspective, knowledge and opinion of their conversational partner (Bebko and McKinnon, 1993). Each item is rated on a scale with five options: past this level, yes, emerging, not yet, or unsure. Up to 18 points are available for form, 24 for Content, 22 for Reference, 22 for Cohesion, and 26 for Use. We combined the scores on the five sections to give an aggregate score (out of a possible 112 points). The LPP-2 takes approximately 15 min to complete and has been shown to have good concurrent validity with language measures used with both deaf and hearing children (Bebko et al., 2003).

#### Non-verbal reasoning task

Finally, the *Matrix Reasoning* subtest of the *Wechsler Abbreviated Scale of Intelligence* (WASI; Wechsler, 1999) was also administered as a control measure. Matrix reasoning is a performance IQ assessment of non-verbal fluid ability. The child is presented with a pattern with a missing section and is instructed to select the correct response from five potential choices. The starting and stopping points are determined by the participant's age, and the matrices become increasingly difficult to solve. The test begins with two practice items to ensure the child has understood the task. The test is terminated when four successive answers, or four out of five successive answers, are incorrect.

### Procedure

Prior to data collection, written parental consent was obtained and the LPP-2 questionnaire was also completed by parents. (For three children, all in the deaf group, the LPP-2 was completed instead by the child's teachers). Face-to-face consent was obtained from the children at the start of the testing session. The children were tested individually in a quiet room, either at school or at home, in a session lasting 35–45 min. (The approximate timings for each test were: Corsi blocks—5 min; Odd one out task—5 to 10 min; Narrative—5 to 10 min; Vocabulary—10 min; WASI matrix reasoning—10 min). The entire session was videotaped. Testing of the deaf children was carried out by an adult hearing native user of BSL, who is highly experienced in communicating with deaf children. The hearing children were also tested by this adult and by three additional trained hearing experimenters. Standardized test instructions (translated into BSL) were used for all of the tests. As mentioned earlier, the tasks required minimal verbal/signed instruction, and sufficient practice trials were included to ensure understanding of the task requirements. It was ensured that lighting conditions were good and that children could see the experimenter clearly to view lip movements. The tests were administered in the same order for all participants to ensure that possible test-order effects would be consistent across groups.

## Results

Data were missing from one hearing child for the BSL Narrative Production test, and from five deaf and two hearing children for LPP-2. Otherwise the dataset was complete. We present three sets of analyses. First, we compare the entire group of deaf children to the group of hearing children on all language and working memory measures. Secondly, we split the deaf group according to language experience into native and non-native signer groups, and compare them to the hearing children on all language and working memory measures. Finally, we investigate whether language scores predict working memory scores in the deaf and hearing children considered together.

### Comparison of deaf vs. hearing groups

Raw scores for the deaf and hearing groups on the language and working memory tasks are presented in Table 3. A series of independent samples *t*-tests was carried out to test for group differences. Because the groups did not differ for age and WASI matrix reasoning score, those factors were not controlled for in this analysis.

**Table 3:** **Mean (standard deviation) raw scores for the language and working memory measures, for the deaf and hearing groups**.

	**Measure (highest possible score)**	**Deaf children (*N* = 27)**		**Hearing children (*N* = 28)**	
		**Mean**	**(*SD*)**	**Mean**	**(*SD*)**
Working memory	Spatial span: forwards (16)	6.22	(1.55)	6.75	(1.62)
	Spatial span: backwards (16)	4.93^*^	(1.98)	6.18	(1.98)
	Odd one out span (24)	8.85^*^	(4.03)	12.29	(5.45)
Language	Expressive one word picture vocabulary test (155)	64.44^***^	(15.76)	92.14	(14.06)
	BSL narrative assessment: content (16)	11.22	(3.06)	10.52	(3.38)
	BSL narrative assessment: structure (12)	9.37	(2.02)	9.26	(2.23)
	Language proficiency profile-2 (112)	97.27^**^	(15.86)	108.58	(5.52)

*The deaf group scores significantly lower than the hearing group*:

* *p < 0.05*,

** *p < 0.01*,

*** *p < 0.001*.

For the working memory tasks, the hearing group significantly outscored the deaf group on two measures: Spatial Span Backward, *t*_(53)_ = 2.345, *p* = 0.023, and Odd One Out, *t*_(53)_ = 2.650, *p* = 0.011. There were no group differences on the Spatial Span Forward task, *t*_(53)_ = 1.231, *p* = 0.224.

For the language tasks, the hearing group significantly outscored the deaf group on two measures: the Expressive One Word Picture Vocabulary Test, *t*_(53)_ = 6.883, *p* < 0.001, and the Language Proficiency Profile, *t*_(46)_ = 3.401, *p* = 0.001. There were, however, no group differences for BSL Narrative: Content, *t*_(52)_ = 0.803, *p* = 0.426, and BSL Narrative: Structure, *t*_(52)_ = 0.193, *p* = 0.849. Overall, therefore, where group differences were found on language and working memory measures, they favored the hearing group.

### Comparison of native signer and non-native signer vs. hearing groups

Table 4 presents the results of the language and working memory tasks for the three groups separately. Because the non-native signers were significantly older than the native signers, and because the non-native signers scored lower on the WASI matrix reasoning than both the native signers and the hearing group, we investigated group differences using ANCOVAs wherein we controlled for age and WASI score. Within each ANCOVA we carried out *post hoc* comparisons for group, using the Sidak correction to adjust for multiple comparisons.

**Table 4:** **Estimated marginal means (standard error) for the language and working memory measures (controlling for age and WASI T-score), for the deaf native signer, deaf non-native signer, and hearing groups**.

	**Measure**	**Deaf native signers (*N* = 8)**		**Deaf non-native signers (*N* = 19)**		**Hearing children (*N* = 28)**	
		**Mean**	**(*SE*)**	**Mean**	**(*SE*)**	**Mean**	**(*SE*)**
Working memory	Spatial span: forwards	7.00	(0.55)	5.82	(0.36)	6.80	(0.27)
	Spatial span: backwards	5.79	(0.70)	4.60^*^	(0.45)	6.16	(0.35)
	Odd one out span	10.43	(1.57)	8.40^*^	(1.03)	12.14	(0.78)
Language	Expressive one Word picture Vocabulary test	76.00^**^	(4.69)	59.90^***^	(3.06)	91.93	(2.32)
	BSL narrative assessment: content	11.45	(1.17)	11.23	(0.76)	10.44	(0.59)
	BSL narrative assessment: structure	9.73	(0.78)	9.28	(0.52)	9.22	(0.40)
	Language proficiency profile-2	110.46	(4.81)	91.93^***^	(2.93)	108.82	(2.11)

Group scores significantly lower than the hearing group:

* *p < 0.05*,

** *p < 0.01*,

*** *p < 0.001*.

For the working memory tasks there was a significant effect of group for the Spatial Span Backward task, *F*_(2, 54)_ = 3.449, *p* = 0.040. *Post hoc* tests revealed just one significant group difference: the non-native signers scored significantly more poorly than the hearing group, *p* = 0.034. Likewise, for the Odd One Out task, there was a significant effect of group, *F*_(2, 54)_ = 4.187, *p* = 0.021, with the only group difference being between the non-native signers and the hearing group, *p* = 0.020. There were no group differences for the Spatial Span Forward task, *F*_(2, 54)_ = 2.474, *p* = 0.094.

For the language tasks, the Expressive One Word Picture Vocabulary Test demonstrated a highly significant effect of group, *F*_(2, 54)_ = 34.829, *p* < 0.001, and this was driven by all three groups being significantly different from one another: non-native signer < native signer, *p* = 0.029, non-native signer < hearing, *p* = 0.001, and native signer < hearing, *p* = 0.009. For the Language Proficiency Profile, there was also a significant effect of group, *F*_(2, 47)_ = 10.688, *p* = 0.001, with the non-native signer group scoring significantly lower than both the native signer group, *p* = 0.011, and the hearing group, *p* < 0.001. There were no significant differences in scores between the native signer and hearing groups. As before, there were no group differences for scores on the BSL Narrative Test: Content, *F*_(2, 53)_ = 0.542, *p* = 0.585, and BSL Narrative Test: Structure, *F*_(2, 53)_ = 0.174, *p* = 0.841.

### Using language scores to predict working memory scores

To explore the contribution of language to working memory test scores, a set of multiple regression analyses was carried out across all participants to predict scores on each working memory task. Expressive One Word Picture Vocabulary Test and Language Proficiency Profile scores were used as predictors. (The scores for content and structure in the BSL Narrative Production Test were not used because they had shown no group differences.) Age and WASI matrix reasoning T-scores were used as control predictors, and were entered into the model in a first step.

For the Spatial Span Backward task, a model with just age and WASI score entered as predictors of span scores was significant, *F*_(2, 47)_ = 4.167, *p* = 0.022, adjusted *R*^2^ = 0.119. Both age (β = 0.397, *t* = 2.680, *p* = 0.010) and WASI score (β = 0.299, *t* = 2.016, *p* = 0.050) were unique predictors. When the two language measures were added, the model was a better fit, *F*_(4, 47)_ = 4.465, *p* = 0.004, adjusted *R*^2^ = 0.228. In this new model, both age (β = 0.273, *t* = 1.772, *p* = 0.083) and WASI score (β = 0.174, *t* = 1.196, *p* = 0.238) lost their unique predictive power. Expressive One Word Picture Vocabulary score was a significant unique predictor (β = 0.379, *t* = 2.208, *p* = 0.033), but the Language Proficiency Profile score was not (β = 0.029, *t* = 0.176, *p* = 0.861).

The Odd One Out task showed a similar pattern to the Spatial Span Backward task. Age and WASI score entered together into the model were significant predictors of span scores, *F*_(2, 47)_ = 8.192, *p* = 0.001, adjusted *R*^2^ = 0.234. Both age (β = 0.497, *t* = 3.596, *p* = 0.001) and WASI score (β = 0.427, *t* = 3.092, *p* = 0.003) were unique predictors. When the two language measures were added, the model showed an excellent fit, *F*_(4, 47)_ = 11.368, *p* < 0.001, and explained almost half of the variance in complex span (adjusted *R*^2^ = 0.469). Age (β = 0.329, *t* = 2.576, *p* = 0.014) and WASI score (β = 0.260, *t* = 2.149, *p* = 0.037) remained significant predictors. Expressive One Word Picture Vocabulary score was also a significant unique predictor (β = 0.510, *t* = 3.588, *p* = 0.001), but the Language Proficiency Profile score was not (β = 0.035, *t* = 0.261, *p* = 0.795).

Finally, for the Spatial Span Forward task, age and WASI score entered together into the model were significant predictors of span scores, *F*_(2, 47)_ = 6.456, *p* = 0.003, adjusted *R*^2^ = 0.188. Only age was a unique predictor (β = 0.495, *t* = 3.480, *p* = 0.001). WASI score was not a unique predictor, (β = 0.071, *t* = 0.501, *p* = 0.619). Adding the two language measures improved the model's fit, *F*_(4, 47)_ = 5.746, *p* = 0.001, adjusted *R*^2^ = 0.288. Age remained a significant unique predictor (β = 0.396, *t* = 2.677, *p* = 0.010), but WASI score again was not (β = −0.041, *t* = −0.294, *p* = 0.770). Neither Expressive One Word Picture Vocabulary score (β = 0.318, *t* = 1.933, *p* = 0.060) nor Language Proficiency Profile score (β = 0.086, *t* = 0.553, *p* = 0.583) were unique predictors of Spatial Span Forward score.

### Summary of results

For the Expressive One Word Picture Vocabulary Test we found significant group differences: the deaf group as a whole, and the native signer and non-native signer groups separately, scored more poorly than the hearing group. Furthermore, the non-native signers scored significantly lower than the native signers. For the Language Proficiency Profile, the pattern was a little different: the group difference between the deaf and hearing groups appeared to be driven by the poor performance of the non-native signer group.

The two executive-loaded working memory measures, the Spatial Span Backward and the Odd One Out Task, patterned like the Language Proficiency Profile: the group of deaf children as a whole and the non-native signer group separately scored lower than the hearing children, but the native signer group did not. For these two working memory measures, vocabulary as measured by the Expressive One Word Picture Vocabulary Test was a significant predictor of scores, beyond age and WASI matrix reasoning score, revealing an association between language and executive-loaded working memory.

However, for the BSL Narrative Production Test, scores for Narrative and Content revealed no group differences—the group of deaf children as a whole, and the two separate groups of deaf native and deaf non-native signers, scored at the same level as the hearing group. Similarly, for one of the working memory measures, the Spatial Span Forward task, we found no group differences. Thus, it is not inevitable that language and working memory performance is worse in deaf children compared to hearing—it depends on the nature of the task.

## Discussion

In this study, we investigated the relationship between language and working memory by comparing three groups of children with different language experiences: hearing children, deaf native signers, and deaf non-native signers. These three groups allowed us to tease apart the impact of the quality of auditory experience vs. the impact of reduced language experience on working memory. If disturbances to auditory experience cause poor working memory, then both deaf groups should have performed worse than the hearing group. If it is language experience rather than deafness that impacts on working memory, then deaf native signers should have patterned like hearing children and both groups should have performed better than non-native signers. Furthermore, scores on language tasks should have correlated with working memory scores.

Our findings are consistent with this latter hypothesis that language experience, but not deafness *per se*, impacts on non-verbal, executive-loaded working memory. Although our group of deaf children as a whole performed more poorly than an age-matched group of hearing children on the two tasks that involved executive-loaded working memory (the Spatial Span Backward and the Odd One Out Tasks), this poor performance was driven by those deaf children who had had delayed and reduced language exposure by not having received signed language input from birth. The small subset of children (*n* = 8) who had learnt BSL under “native” language-learning conditions, i.e., from deaf signing parents, and who had rich language interactions throughout their childhood with family members, friends and at school, did not differ from the hearing group in their working memory scores. We do of course need to be cautious in our interpretation: our group of deaf native signers was small, as this is a rare population. Indeed, small sample sizes are prevalent in experimental studies of native signers' working memory (e.g. *n* = 6 in Wang and Napier, 2013; *n* = 8 in Krakow and Hanson, 1985; *n* = 11 in Wilson and Emmorey, 2006). Finally in our study, vocabulary, as indexed by the Expressive One Word Picture Vocabulary Test, was a strong predictor of scores on both executive-loaded working memory tasks when all children were considered together.

As discussed in the introduction, teasing apart causal relations between two variables over developmental time is not straightforward. For example, working memory has been extensively investigated in children with Specific Language Impairment (SLI). It has been argued that poor language directly impacts on working memory in children with SLI (van der Lely and Howard, 1993), but others have argued otherwise. In a more recent study Henry et al. (2012) administered the same non-verbal Odd One Out task as we used in our study (Henry, 2001) and a verbal working memory task, Listening Recall (Working Memory Test Battery for Children, Pickering and Gathercole, 2001). Henry et al. (2012) found that groups of children with poor language [both normal IQ (i.e., SLI) and low IQ] scored lower than typically developing children on both the verbal and non-verbal working memory tasks. In particular, performance on these tasks remained lower for the SLI group even when verbal IQ was entered in the regression analyses. Henry et al. (2012) conclude that their results are consistent with SLI being caused by a domain-general impairment rather than by an impairment specific to language (see also Ullman and Pierpont, 2005). However, these issues are difficult to tease apart in a population that is heterogeneous with respect to the severity and profile of language difficulties, and where it is possible that deficits of both domain-general and domain-specific (i.e., language) origin co-occur in the same child.

In contrast, the current study involved groups of children where language experience is affected in two, separable, ways: by deafness, and by parental language skills. In contrast to SLI, where the cause of language impairment is inherent and neurological, and therefore may or may not involve other cognitive functions, deafness directly affects children's access to spoken language but would not be expected to directly affect working memory. Nevertheless, poorer performance on working memory tasks has been noted in deaf individuals. By investigating a deaf population that includes both native and non-native signers, we can begin to explore whether concurrent impairments in working memory are linked more closely with language ability or with deafness *per se*. By avoiding tasks that require auditory instructions, stimuli and responses, we removed the immediate disadvantage that deaf children face when being compared to hearing children. Our results indicate that when children do not have adequate exposure to a native language—regardless of its modality—this has consequences for the development of wider cognitive skills (see Campbell et al., 2014, for a discussion of the neurocognitive consequences of late age of exposure).

However, we do need to be careful when considering the data in our study—does the association between language and non-verbal working memory arise because language is mediating performance on working memory tasks concurrently, or because language has had a developmental effect on working memory up until this point in the child's life? We have been particularly careful to choose tasks that we think do not benefit from verbal mediation, and therefore, the measures should not contribute to poorer performance in this way. Nevertheless, we are aware that the nature of verbal mediation in visual tasks is not fully understood, and that research is only just beginning to explore this in children with atypical development. For example, Lidstone et al. (2012) showed that children with and without SLI were equally affected by a verbal suppression task during the Tower of London (executive memory planning task) despite the fact that children with SLI performed more poorly on the task overall. Ideally, longitudinal and training studies would help to elucidate this issue.

Furthermore, our results indicate that of the four language measures used—the Language Proficiency Profile, Expressive One Word Picture Vocabulary Test, BSL Narrative: Content and BSL Narrative: Structure—it was the Expressive One Word Picture Vocabulary Test that predicted executive-loaded working memory scores. One interpretation is that the ability to name stimuli and describe them during such tasks allows verbal mediation and draws on vocabulary skills. However, we did not have a measure of syntax, which might also be involved in verbal mediation. The grammatical structure of BSL and English is very different and not easily directly comparable, and whether syntax could be a predictor of working memory scores in our participants remains to be tested.

Finally, although we have interpreted our results as indicating that language experience directly impacts working memory, there are other differences in the developmental experience of native and non-native signers apart from language exposure that might be at play here. These include parental attachment, attention-getting strategies and social-cognitive development, among others (Marschark and Hauser, 2012). It is possible that some or all of these factors work alongside language exposure to influence working memory development. More research with larger numbers of native signers is required to fully understand these relationships.

Despite these caveats in the interpretation of our results, we argue that contrasting deaf children who grow up in optimal and suboptimal language-learning environments offers a valuable method for understanding the relationship between language and working memory. When the majority of deaf children start to develop language, they experience suboptimal conditions because the language context is predominantly oral. When this “adverse” condition is not present (i.e., when the child's deaf parent signs with them) we see a very different picture that can inform both theory and clinical practice. In particular, deafness might not be, in itself, a barrier to the development of good working memory abilities. With early exposure to an accessible sign language deaf children can demonstrate comparable skills to their hearing peers in this crucial domain. We would not wish for our results to be taken as indicating that early exposure to sign language does not help deaf children from *hearing* families—indeed, we would argue the opposite. An obvious implication for interventions with deaf children of hearing parents is for accessible language exposure to be provided early enough and in contexts where it can enhance or interact with working memory skills. A next step is to understand which aspects of language (e.g., communicative practices between interlocutors or more particular components of language such as vocabulary or syntax) are more closely involved in enabling the full development of working memory. We still lack sufficient information about the timing, amount and quality of sign language exposure that might be necessary to support age-appropriate cognitive development, and we hope to see more future research that investigates those relationships.

### Conflict of interest statement

The authors declare that the research was conducted in the absence of any commercial or financial relationships that could be construed as a potential conflict of interest.
